# Protective Role of Antioxidant Huskless Barley Extracts on TNF-*α*-Induced Endothelial Dysfunction in Human Vascular Endothelial Cells

**DOI:** 10.1155/2018/3846029

**Published:** 2018-05-10

**Authors:** Zhanghua Liao, Haoran Cai, Zekun Xu, Jing Wang, Chen Qiu, Jing Xie, Wuyang Huang, Zhongquan Sui

**Affiliations:** ^1^Department of Food Science and Engineering, School of Agriculture and Biology, Shanghai Jiao Tong University, Shanghai 200240, China; ^2^College of Chemical Engineering, Nanjing Forestry University, No. 159 Longpan Road, Nanjing 210037, China; ^3^Institute of Farm Product Processing, Jiangsu Academy of Agricultural Sciences, Nanjing 210014, China; ^4^Jiangsu Key Laboratory for Horticultural Crop Genetic Improvement, Jiangsu Academy of Agricultural Sciences, Nanjing 210014, China

## Abstract

Oxidative stress and inflammation are considered as two key factors that contribute to the development of atherosclerosis. This study was to investigate the antioxidant capacity of huskless barley and to explore its protective functions through the regulation of the antioxidant defense and inflammatory response in human umbilical vein endothelial cells (HUVEC). The oxygen radical absorbance capacity (ORAC), ferric-reducing antioxidant power (FRAP), and 2,2-azino-bis(3-ethylbenzothiazoline-6-sulfonic acid) diammonium salt (ABTS) scavenging capacity of water and alkali extracts of the polysaccharides from nine huskless barley varieties were investigated *in vitro*. The antioxidant properties of the alkaline extracts were more pronounced than those of the water extracts. The results from the cell model showed that pretreatment of HUVEC with the water or alkaline extracts of the polysaccharides from the huskless barley cultivars QHH and NLGL decreased the levels of reactive oxygen species (ROS), monocyte chemotactic protein 1 (MCP-1), and vascular cell adhesion molecule 1 (VCAM-1) but increased the level of superoxide dismutase (SOD) and maintained cell viability. Huskless barley polysaccharide extracts exhibited the vasodilatory effect of inhibiting angiotensin-converting enzyme (ACE) production. These discoveries revealed the potent protective functions of barley in oxidative damage and a potential role for barley in preventing chronic inflammation in cardiovascular diseases.

## 1. Introduction

The vascular endothelium is the epithelial tissue that lines the blood vessels. This tissue serves as a regulator of the vascular wall function. Changes in its structure and function form the common pathological basis of cardiovascular diseases. Endothelial dysfunction is considered as an important step in the development of atherosclerosis (AS), which is associated with an inflammatory response and the increased interaction of platelets and monocytes with the vessel wall [1, 2]. A number of inflammatory mediators are released by the endothelial cells in response to localized injury or trauma. The primary inflammatory mediators are categorized into four main groups: lipids, plasma enzymes, cytokines, and chemokines [3]. Among these mediators, tumor necrosis factor-alpha (TNF-*α*), a prototypical proinflammatory cytokine that is found in atherosclerotic lesions, can exert direct effects on vascular endothelial cells to induce the expression of adhesion molecules such as vascular cell adhesion molecule-1 (VCAM-1) in leukocytes and other inflammatory cells. Accordingly, TNF-*α* facilitates the progression of atherosclerotic cardiovascular disease [4]. Considerable evidence has shown that antioxidative, anti-inflammatory, and antihypertensive effects may be highly correlated with the inhibition of AS [5, 6].

Reactive oxygen species (ROS) may reduce the availability of endothelial nitric oxide (NO). Additionally, the reaction between ROS and NO forms a more reactive peroxide, nitroso peroxide, which further augments endothelial dysfunction [7]. Moreover, increased levels of vascular superoxide inhibit the vascular extracellular superoxide dismutase (ecSOD) activity, which leads to the production of more superoxide, inhibits the function of NO, and causes endothelial damage [8]. Therefore, antioxidants can reduce oxidative stress by directly scavenging ROS, increasing the SOD activity, and regulating NO to improve endothelial function. Together, these processes have the effects of preventing and treating hypertension.

Grains, cereals, vegetables, and fruits are good sources of antioxidants including polyphenols, flavonoids, and polysaccharides [9, 10]. As a major cereal crop that is used extensively as an industrial commodity and in fermentation products, barley is a functional cereal that is rich in dietary fiber. Dietary fiber (mainly non-starch polysaccharides) plays an important role in the health benefits associated with whole grain consumption, which has increasingly attracted the interest of scientists [11]. The physiological and functional properties of dietary fiber depend on the food source, extraction method, chemical composition, structure, and particle size [12, 13]. Knutsen and Holtekjolen reported that the water extracts of the polysaccharides consisted mostly of beta-glucans whereas the alkaline extracts consisted of arabinoxylans (AX) [14]. The carbohydrate composition in different barley varieties differs considerably; thus, the selection of the variety is also important.

Although it is widely recognized that dietary fiber possesses considerable antioxidant capacity, the antioxidative capacity of barley remains unknown. In the present study, we validated the antioxidant capacity of water and alkali extracts of the polysaccharides from nine huskless barley varieties *in vitro* by measuring the oxygen radical absorbance capacity (ORAC), ferric-reducing antioxidant power (FRAP), and 2,2-azino-bis(3-ethylbenzothiazoline-6-sulfonic acid) diammonium salt (ABTS) scavenging capacity. Moreover, the protective role of barley in cells was studied. We hypothesized that huskless barley extracts may slow the process of atheroma formation by inhibiting TNF-*α*-induced cell adhesion through the suppression of superoxide anion production in human umbilical vein endothelial cells (HUVEC). The purpose of this study was to explore the inhibitory effects of the polysaccharide extracts of barley on the vascular inflammatory process in TNF-*α*-induced HUVEC and to elucidate the mechanism of the antioxidative action. We conducted research on the protection of vascular endothelium by investigating the expression of several molecules in TNF-*α*-induced HUVEC that had been pretreated with polysaccharide extracts from two representative huskless barley cultivars. Moreover, the results from two extraction methods (water and alkaline) were compared to help understand and analyze the active component in extracts.

## 2. Materials and Methods

### 2.1. Materials and Chemicals

Nine different cultivars of huskless barley were obtained as gifts from Diqing Shangri-La Huskless Barley Development Co. Ltd., Yunnan, China. Their codes and names are listed as follows: (a) Black and white huskless barley 80 days (BW80) from Xianggelila; (b) JiuGe (JG) from Xianggelila; (c) DiQing 3 (DQ3) in the winter season; (d) long and black huskless barley (LB) from Xianggelila; (e) YunQing 2 (YQ2); (f) GuiBaDingGeNa (GBDGN) from Deqin; (g) short and white huskless barley (SW) from Xianggelila; (h) QingHaiHuang (QHH) in the winter season; and (i) NanLongGeNa (NLGN) from Yanmen in Deqin.

A human umbilical vein endothelial cell (HUVEC) line was purchased from Zhongqiao Xinzhou Biological Technology Co. Ltd. (Shanghai, China). Dulbecco's phosphate-buffered saline (PBS), M199 medium, 2,2′-azobis(2-methylpropionamide) dihydrochloride (AAPH), 2,2-azino-bis(3-ethylbenzothiazoline-6-sulfonic acid) diammonium salt (ABTS), 3-(4,5-dimethyl-2-thiazolyl)-2,5-diphenyl-2-H-tetrazolium bromide (MTT), and TNF-*α* were obtained from Sigma Chemical Co. Ltd. (Nanjing, China). Trolox (6-hydroxy-2,5,7,8-tetramethylchromate-2-carboxylic acid) was obtained from Acros Organics (Shanghai, China). A reactive oxygen species (ROS) assay kit was obtained from the Beyotime Institute of Biotechnology (Shanghai, China). Streptomycin and penicillin were purchased from Life Technologies (Shanghai, China). Enzyme-linked immunosorbent assay (ELISA) kits for superoxide dismutase (SOD), monocyte chemotactic protein 1 (MCP-1), vascular cell adhesion molecule 1 (VCAM-1), and human angiotensin-converting enzyme (ACE) were purchased from Boster Biotechnology Inc. (Wuhan, China). All chemicals were analytical grade.

### 2.2. Extractions of Huskless Barley Polysaccharide

The extraction method was based on the Association of Official Analytical Chemists (AOAC) 2009.01 protocol with minor modifications. Briefly, 10 g of a milled defatted sample, in duplicate, was subjected to sequential enzymatic digestion by heat-stable *α*-amylase (50 *μ*L, 80°C water bath for 1 h), papain (15 mg, 60°C water bath for 1 h), and glucoamylase (400 *μ*L, 60°C water bath for 1 h) to remove the starch and protein. The slurry was incubated twice with distilled water at 80°C for 3 h. After centrifugation at 3000 rpm for 30 min, the supernatant was combined with the water washings of the residue, and this mixture was precipitated in ethanol (100%, 3 volumes) with continuous stirring. The precipitate was washed with acetone, dried under nitrogen, and labeled as hot water-extracted polysaccharide (WE). To prepare the alkaline extracts of the polysaccharides, the residue was washed twice with NaOH (0.5 mol/L, 40 mL) in a 70°C water bath for 2 h. The alkaline washings were centrifuged at 3000 rpm for 30 min. The mixture was neutralized with 0.5 M HCl. The supernatant was dialyzed with three times its volume of distilled water and precipitated in ethanol (100%, 3 volumes) with continuous stirring. The precipitate was washed with acetone, dried under nitrogen, and labeled as alkaline-extracted polysaccharide (AE).

### 2.3. 2,2-Azino-bis(3-ethylbenzothiazoline-6-sulfonic Acid) Diammonium Salt-Scavenging Capacity Assay

The ABTS free radical-scavenging activity of each extracted sample was determined according to the method described by Arts et al. [15]. Various levels (4, 2, 1, 0.5, 0.25, and 0.125 g/L) of the samples and a standard solution of Trolox in DMSO were prepared and assayed. The absorbance at 734 nm of the resulting oxidized solution was detected. The results were expressed in terms of the Trolox equivalent antioxidant capacity (TEAC), that is, micrograms of Trolox equivalent per gram of dry weight (mg Trolox/g dried weight (DW)). The half maximal inhibitory concentration (IC_50_, g/L) was also determined.

### 2.4. Ferric-Reducing Antioxidant Power Assay

The FRAP assay was conducted using a modified version of the method described by Benzie and Strain [16]. Aliquots of various levels (4, 2, 1, 0.5, 0.25, and 0.125 g/L) of the samples were added to the FRAP reagent. The increase in absorbance at 593 nm was measured. Fresh working solutions of FeSO_4_ were used for calibration. The antioxidant capacity was calculated from the linear calibration curve and expressed as micromoles of FeSO_4_ equivalent per gram of dry weight (*μ*mol FeSO_4_/g DW).

### 2.5. Oxygen Radical Absorbance Capacity Assay

The radical-scavenging activity was assayed using the improved ORAC method as described previously [17]. The fluorescence decay curves of fluorescein in the presence of WE, AE, and Trolox at various concentrations were generated with MikroWin Microplate Data Reduction 2000 (Mikrotek Laborsysteme GmbH, Overath, Germany). The ORAC value was calculated from the slope of the sample equation dividing the slope of the Trolox curve obtained for the same assay. The final ORAC value is expressed as micromoles of Trolox equivalent per gram of dry weight (*μ*mol Trolox equivalent (TE)/g DW).

### 2.6. Endothelial Cell Culture and Treatment

Human umbilical vein endothelial cells are considered a model system for studying the oxidative stress and anti-inflammatory and antihypertension activities in the vasculature. The cells were cultured according to a laboratory protocol. After stabilization in a reduced serum medium for 4 h prior to the beginning of the actual experiment, the cells were treated with water extracts of QHH (WE-QHH), alkaline extracts of QHH (AE-QHH), water extracts of NLGN (WE-NLGN), and alkaline extracts of NLGN (AE-NLGN) (10 mg/L) for 18 h in a separate set of experiments followed by the stimulation with TNF-*α* (10 *μ*g/L). Dimethylsulfoxide (DMSO) was used in the control. At the end of the specified incubation period, the supernatants were collected and stored at −80°C until further analysis of MCP-1 and VCAM-1.

### 2.7. Cell Viability Assay

Human umbilical vein endothelial cells (20000 cells/well) were cultured according to the treatment above after which 20 mL of the stock solution of MTT (5 mg/mL) was added to each well for 4 h. Finally, the incubation medium was removed, and the formazan crystals were dissolved in 150 *μ*L of DMSO. The MTT reduction was measured as the absorbance at 490 nm using a StatFax-2100 Microplate Reader (Awareness Technology Inc., Palm City, FL). The background absorbance of the control wells was subtracted. The analysis was performed in triplicate. Viability (%) = OD (experiment group)/OD (control) × 100%.

### 2.8. Reactive Oxygen Species Assay

A DCFH-DA detection kit (Beyotime Institute of Biotechnology, Shanghai, China) was used to assess the ROS level in HUVEC. The cells were seeded in 6-well plates, treated with the various samples, and incubated for 24 h. After washing the cells with the reduced-serum medium, 10 *μ*mol/L DCFH-DA was added to each well, and the cells were incubated at 37°C for 20 min. The cells were then washed thoroughly with reduced-serum medium to remove the DCFH-DA that did not enter the cells. The cells were collected in 1 mL of PBS after dissociation, and the fluorescence was immediately recorded using a LB 941 TriStar Microplate Reader (Berthold Technologies, Bad Wildbad, Germany) using 485 nm excitation and 535 nm emission filters. The total fluorescence intensity of the cells in each well was recorded, and ROS generation was measured as the fold increase over the untreated control.

### 2.9. Enzyme-Linked Immunosorbent Assay

The levels of SOD, MCP-1, VCAM-1, and ACE in the supernatants were quantified using the solid-phase sandwich ELISA kits. The assay procedures were performed according to the instructions in the kit protocol booklets. The samples were used to conduct a protein assay and were suitably diluted in the standard diluent buffer. The absorbance of the resulting yellow color was measured at 450 nm using a StatFax-2100 Microplate Reader (Awareness Technology Inc., Palm City, FL). The reader was controlled via Hyper Terminal Applet ELISA software.

### 2.10. Statistical Analysis

The data are presented as the mean values ± standard deviation (SD) and were analyzed using the analysis of variance (ANOVA). The data figures were prepared using GraphPad Prism version 5.02 (GraphPad Software Inc., CA, USA). Duncan's multiple range tests (*P* < 0.05) were conducted to analyze their differences. A two-way ANOVA was used to analyze the differences among the alkaline extracts and water extracts of the nine barley varieties. The statistical analyses were performed using SPSS 17.0 for Windows (SPSS Inc., Chicago, IL, USA).

## 3. Results

### 3.1. Antioxidant Capacity of Huskless Barley Polysaccharide Extracts *In Vitro*

**Figure 1:**
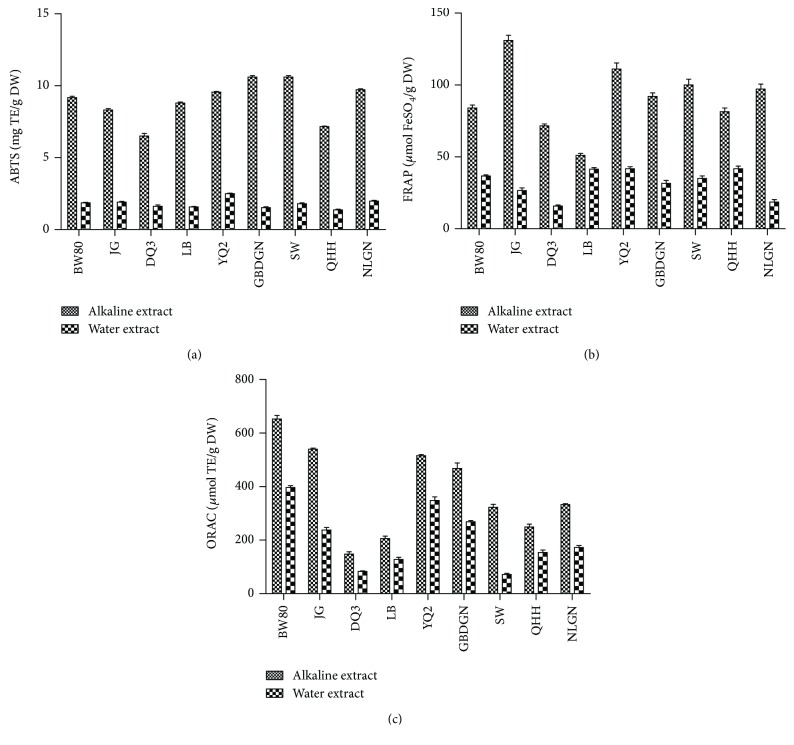
Antioxidant capacity of various huskless Barley extracts *in vitro*. (a) ABTS-scavenging capacity; (b) ferric-reducing antioxidant power (FRAP); and (c) oxygen radical-absorbance capacity (ORAC).

Table 1 shows the results for the antioxidant capacity of the water and alkali extractions from the nine huskless barley varieties *in vitro*, including the ABTS, FRAP, and ORAC data. The IC_50_ values for the AEs in the ABTS radical assay ranged from 1.74 to 2.84 g/L with a mean value of 2.12 g/L. The mean values for the TEAC, FRAP, and ORAC assays were 8.73 mg/g DW, 91.95 *μ*mol/g DW, and 381.39 *μ*mol TE/g DW, respectively. The IC_50_ values for the WEs in the ABTS radical assay ranged from 7.41 to 13.43 g/L with a mean value of 10.59 g/L. The mean values for the TEAC, FRAP, and ORAC assays were 1.79 mg/g DW, 32.61 *μ*mol/g DW, and 251.06 *μ*mol TE/g DW, respectively. There were significant differences between the alkaline and water extracts in the ABTS, FRAP, and ORAC results (*P* < 0.001). Each alkaline extract possessed a much higher antioxidant capacity than the water extract for each huskless barley variety (Figure 1).

The various huskless barley varieties exhibited different antioxidant capacities using the different models. The extracts AE-GBGN and AE-SW had the strongest ABTS-scavenging capacity with the highest TEAC values (both 10.61 mg/g DW) and the lowest IC_50_ values (both 1.74 g/L). The extracts AE-NLGN, AE-YQ2, and AE-BW80 also had high ABTS-scavenging capacities with values over 9.00 mg/g DW TEAC. The extract WE-QHH showed the lowest ABTS-scavenging capacity, but AE-QHH was not the lowest of the nine AEs. The extract AE-JG had the highest FRAP value (131.1 *μ*mol/g DW), followed by AE-YQ2 (111.1 *μ*mol/g DW), AE-SW (100.0 *μ*mol/g DW), AE-NLGN (97.2 *μ*mol/g DW), and AE-GBGN (92.1 *μ*mol/g DW). Both the AE and WE from BW80 had the best oxygen radical-absorbance capacity. The extract WE-BW80 was also superior to the AEs of most varieties. The ORAC values for AE-BW80 and WE-BW80 were 652.45 and 396.57 *μ*mol TE/g DW, respectively. Moreover, AE-JG and AE-YQ2 had high oxygen radical-absorbance capacities, with ORAC values of over than 500 *μ*mol TE/g DW. The extracts of QHH and NLGL showed moderate antioxidant activities *in vitro*, and their AEs and WEs were used to further study the mechanism of antioxidative action in the cell model.

### 3.2. Effect of Huskless Barley Polysaccharide Extracts on Cell Viability

**Figure 2:**
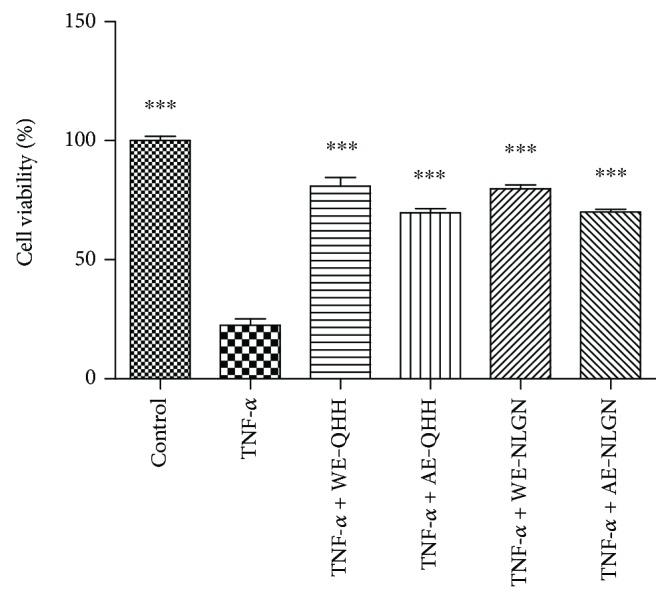
Effects of the water and alkaline extracts from QHH and NLGN on cell viability. ∗∗∗ indicates *P* < 0.001 versus the TNF-*α* group.

To assess whether the inhibitory effect of barley on the stimulation by TNF-*α* could be attributed to its effect on cell viability, we examined the cytotoxic effect of water and alkaline extracts of the huskless barley polysaccharides using the MTT assay, which provides rapid and precise results for cellular growth (Figure 2). A concentration of 10 *μ*g/L TNF-*α* substantially decreased the cell viability of HUVEC from 100% to 22.56% (*P* < 0.001) whereas the huskless barley extract pretreatments effectively reduced the effect of TNF-*α*, facilitating cell proliferation and vascular reproduction. The viabilities of the cells treated with WE-QHH, AE-QHH, WE-NLGN, and AE-NLGN were 80.91%, 69.66%, 79.76%, and 70.04%, respectively. The water extracts seemed to be more compatible with cellular growth than the alkaline extracts.

### 3.3. Effects of Huskless Barley Polysaccharide Extracts on SOD and ROS in Cells

**Figure 3:**
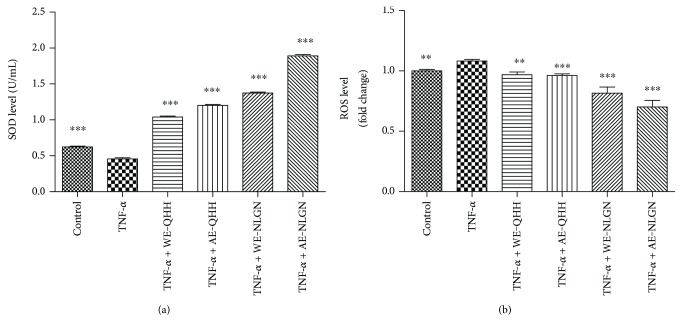
Effects of water and alkaline extracts from QHH and NLGN on (a) SOD expression and (b) ROS levels in the TNF-*α*-induced HUVEC. ∗∗ and ∗∗∗ indicate *P* < 0.01 and *P* < 0.001, respectively, versus the TNF-*α* group.

The addition of TNF-*α* (10 *μ*g/L) greatly decreased the level of SOD in the HUVEC (Figure 3(a)). However, when treated with the dietary fiber extracts from barley, the HUVEC presented higher levels of SOD production. Compared to the HUVEC that were stimulated with TNF-*α* alone, the SOD production of the barley-treated HUVEC was found to be 2.27, 2.62, 3.01, and 4.14 times higher (*P* < 0.001). The addition of the extracts from the huskless barley decreased the ROS values in the endothelial cells. The water and alkaline extracts from QHH resulted in 10.5% and 11.2% inhibition of ROS, respectively. The presence of NLGL decreased the ROS production by 24.7% (WE) and 35.2% (AE) (Figure 3(b)). Similar to the antioxidant capacity *in vitro*, the alkaline extracts seemed to have more pronounced antioxidant activity in cells than the water extracts, and NLGL possessed better antioxidant activity than QHH.

### 3.4. Effects of Huskless Barley Polysaccharide Extracts on the TNF-*α*-Induced MCP-1 and VCAM-1 Protein Expression in Cells

**Figure 4:**
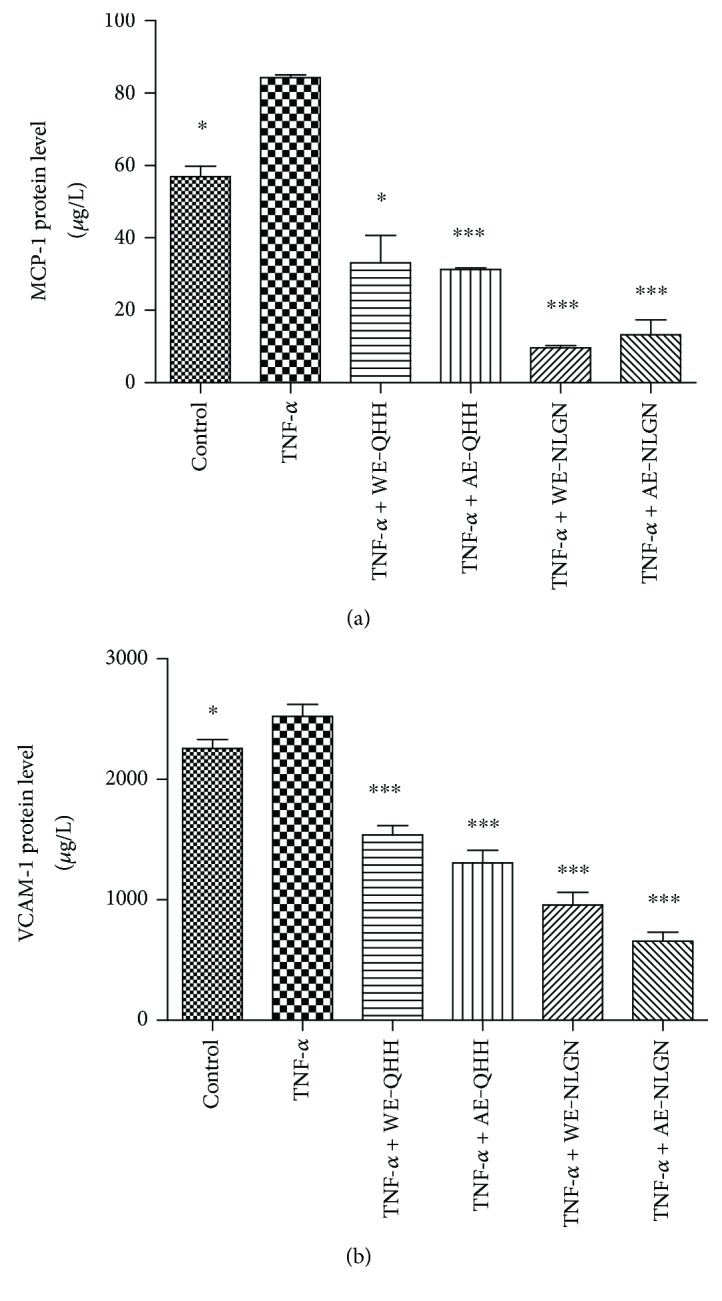
Effects of the water and alkaline extracts from QHH and NLGN on (a) MCP-1 and (b) VCAM-1 expression in the TNF-*α*-induced HUVEC. ∗ and ∗∗∗ indicate *P* < 0.05 and *P* < 0.001, respectively, versus the TNF-*α* group.

Figures 4(a) and 4(b) show that 10 *μ*g/L of TNF-*α* significantly increased the production of MCP-1 and VCAM-1 in the endothelial cells (*P* < 0.05) compared to the control group. Both types of polysaccharide extracts from the two cultivars of huskless barley affected the protein levels of endothelial MCP-1. The WE-QHH, AE-QHH, WE-NLGN, and AE-NLGN extracts inhibited the TNF-*α*-induced MCP-1 levels by 60%, 62.9%, 88.6%, and 84.3%, respectively (*P* < 0.001). Similar to the effects on the MCP-1 protein, the levels of the VCAM-1 adhesion molecule in the cells that were pretreated with WE-QHH, AE-QHH, WE-NLGN, and AE-NLGN decreased by 39%, 48%, 62%, and 74%, respectively, compared with the TNF-*α*-stimulated group (*P* < 0.001). The extract of NLGL also possessed better anti-inflammatory activity than that of QHH. The difference between the effects of the water extracts and alkaline extracts on the MCP-1 protein expression was not significant. However, the alkaline extract inhibited the VCAM-1 protein expression by the cells more than the water extract for QHH and NLGL (*P* < 0.05), which was consistent with their antioxidant capacity.

### 3.5. Effects of Huskless Barley Polysaccharide Extracts on the TNF-*α*-Induced ACE Protein Expression in Cells

**Figure 5:**
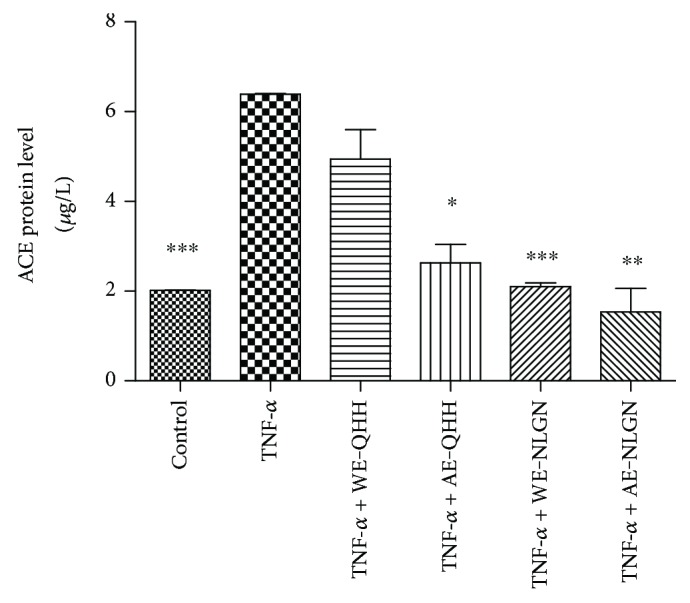
Effects of the water and alkaline extracts from QHH and NLGN on ACE expression in the TNF-*α*-induced HUVEC. ∗, ∗∗, and ∗∗∗ indicate *P* < 0.05, *P* < 0.01, and *P* < 0.001, respectively, versus the TNF-*α* group.

We investigated the effect on ACE production (Figure 5) in our study. When treated with TNF-*α* (10 *μ*g/L), the level of ACE in the endothelial cells increased significantly (*P* < 0.001). The polysaccharide extracts of huskless barley caused a significant reduction in the TNF-*α*-induced levels of ACE in the endothelial cells (*P* < 0.001). The extracts WE-QHH, AE-QHH, WE-NLGN, and AE-NLGN inhibited the ACE levels by 23%, 59%, 67%, and 76%, respectively, compared with the TNF-*α*-induced group. Similarly, the inhibitory effects of the alkaline ACE extracts were more pronounced than those of the water extracts, and the effects of NLGL were more pronounced than those of QHH.

## 4. Discussion

Huskless barley has attracted the attention of researchers and food processors in recent years for its potential health benefits. Huskless barley is recognized as a functional grain because it contains high levels of polysaccharides and phytochemicals [18]. In the present study, nine huskless barley varieties exhibited good antioxidant capacity *in vitro* using various methods (ABTS, FRAP, and ORAC). The huskless barley varieties exhibited different antioxidant capacities due to their different polysaccharide and phytochemical compositions. Djurle et al. reported differences in the carbohydrate composition in six different varieties of barley kernels [19]. For example, the KVL 301 variety had much lower extractability (76%) of mixed-linkage (1 → 3), (1 → 4)-beta-D-glucan after extrusion than the other varieties (91–98%). The antioxidant properties of the alkaline extracts were more pronounced than those of water extracts *in vitro* and in cells. The different constituents of the water extracts (beta-glucans) and alkali extracts (arabinoxylans) might contribute to the differences in their bioactivities [14]. Sawicki et al. considered that in addition to the dietary fiber, other ingredients in the outer bran layer, including phenolic acids, alkylresorcinols, lignans, phytosterols, and tocols, may also contribute to health benefit outcomes [11]. Zhu et al. found that the extracts from four varieties of dehulled highland barley (*Hordeum vulgare* L.) showed excellent antioxidant activities as determined by ORAC and cellular antioxidant activity (CAA) assays; additionally, these varieties showed potent antiproliferative activity towards HepG2 human liver cancer cells. The bound phenolics make a significant contribution to antioxidant and anticancer activities in this model [20]. The alkaline extracts had higher total phenolic and flavonoid content than the water extracts in our tested barley varieties. For example, each gram of WE-QHH, AE-QHH, WE-NLGN, and AE-NLGN contained 1.28, 4.20, 2.89, and 5.14 mg of gallic acid equivalent, respectively. The total phenolic content of the alkaline extracts was nearly two to three times that of the water extracts, which may be another major reason for superior antioxidant activities of the alkaline extracts. The HPLC profiles of the extracts also showed that phenolic compounds of the alkaline extracts were more than those of the water extracts, in which phenolic acids (e.g., gallic acid, chlorogenic acid, 3,5-dicaffeoylqunic acid, and 4,5-dicaffeoylqunic acid) and flavonoids (e.g., rutin and astragalin) were detected (see the supporting document). The antioxidant properties of the dietary fiber from huskless barley bran also showed that the DPPH (1,1-diphenyl-2-picrylhydrazyl radical 2,2-diphenyl-1-(2,4,6-trinitrophenyl)hydrazyl) radical-scavenging activity and ferric-reducing antioxidant power increased with an increase in the total phenolic content [21].

The nine huskless barley varieties evaluated here exhibited antioxidant capacities *in vitro*, and two representative barley cultivars, QHH and NLGN, showed antioxidant action as indicated by the increases in SOD and decreases in the ROS in the human umbilical vein endothelial cells. Superoxide dismutase is an enzyme that converts deleterious O_2_ into less harmful H_2_O_2_ [22]. Superoxide dismutase is highly crucial for oxidative stress tolerance [23] and is recognized as an antioxidative defense against endothelial damage [24, 25]. Reactive oxygen species have destructive actions on both proteins and DNA and are therefore regarded as pathogenic, resulting in cellular death and arterial disease [26, 27]. Increases in ROS are associated with the accumulation of highly reactive free radicals, which exert deleterious effects. Moreover, overexpression of ROS may cause other side effects such as DNA mutations and genetic instability [28]. Therefore, the capacity of water and alkali extracts of the polysaccharides from huskless barley to increase SOD and to decrease ROS indicates that huskless barley can be used as an agent to alleviate harmful effects and protect the cells from oxidative impairment.

In this study, water and alkaline extracts of the polysaccharides from QHH and NLGN inhibited the vascular inflammatory process in TNF-*α*-induced endothelial cells by reducing the expression of the MCP-1 and VCAM-1 proteins. Cell adhesion molecule MCP-1 accumulates in large quantities during various inflammatory diseases [29, 30]. As a proinflammatory cytokine, TNF-*α* can induce the expression of chemokines, cytokines, and cell adhesion molecules in vascular endothelial cells. Stimulation of MCP-1 gene expression is associated with oxidation-reduction-sensitive mechanisms [31, 32]. Cell adhesion molecule VCAM-1 is also important in the inflammatory responses and plays a significant role in cell adhesion and cell signal transduction [4]. Cybulsky et al. found that an early form of cell lesion was significantly diminished by a low level of VCAM-1, which suggested that VCAM-1 contributed to the initiation of AS [33]. In endothelial cells, this adhesion molecule has been revealed to actively participate in the vital functions of immune surveillance and inflammation and the migration of leukocytes from the blood into tissues [34]. The inhibition of the TNF-*α*-stimulated MCP-1 and VCAM-1 expression indicates that huskless barley might contribute to anti-inflammatory activity.

In addition, WE-QHH, AE-QHH, WE-NLGN, and AE-NLGN exhibited vasodilatory effects by inhibiting the production of ACE. Angiotensin-converting enzyme, a carboxyl-terminal dipeptidyl exopeptidase, indirectly leads to hypertension by causing blood vessels to constrict, which is highly correlated with cardiovascular disease [35]. Its mechanism is the conversion of the decapeptide angiotensin I to the potent vasoconstrictor-octapeptide angiotensin II (Ang II). Several studies have shown that Ang II has crucial proinflammatory actions in the vascular wall, including the production of reactive oxygen species that in turn increase the expression of inflammatory cytokines. The inhibition of ACE reduces the Ang II receptor 1 (AT1) expression in HUVEC, which is thought to be the mechanism by which it decreases adhesion molecule production [36]. Therefore, it is reasonable to conclude that huskless barley possesses the potential to reduce the risk of cardiovascular disease through its antihypertensive effects on the vasculature.

In this study, huskless barley extracts exhibited antioxidant capacity. Alkaline-extracted polysaccharide possessed better antioxidant capacity and higher phenolic content than hot water-extracted polysaccharide. Since polysaccharides usually present as complex insoluble bound esters with phenolic compounds [37], phenolics might contribute part of antioxidant function. However, because of a small quantity of phenolics in polysaccharide extracts and the complexity of the natural crude extracts, it is rather difficult to characterize every compound and elucidate the structure. Further phytochemical analysis and the detailed composition of polysaccharide from huskless barley will require identification by LC-MS and NMR in the future. In addition, the huskless barley extracts were found to protect HUVEC against TNF-*α*-induced oxidative stress. However, the mechanism on antioxidant function of huskless barley extracts was unknown. Previous studies reported that dietary antioxidants could regulate oxidative stress in cells by activating Nrf2 (nuclear factor erythroid 2-related factor-2) or its related genes, such as wheat bran feruloyl oligosaccharides increasing SOD, catalase, and glutathione peroxidases via Nrf2 signalling [38]. Whether huskless barley extracts protect HUVEC against TNF-*α*-induced oxidative stress via Nrf2 signalling or its related genes will require further study in the future.

## 5. Conclusions

The results of the present study showed that the nine huskless barley varieties had good antioxidant capacities *in vitro*, including ABTS-scavenging capacity, ferric-reducing antioxidant power, and oxygen radical-absorbance capacity. The antioxidant properties of the alkaline extracts were more pronounced than those of the water extracts. Two representative huskless barley cultivars, QHH and NLGN, alleviated the negative effects of TNF-*α* by blocking the overexpression of the levels of several key proteins, MCP-1, VCAM-1, and ACE, in the HUVEC. Moreover, these cultivars increased the level of SOD and maintained the cell viability. Thus, these cultivars exerted antioxidant, anti-inflammatory, and antihypertensive effects on vascular endothelial cells and have a potential as potent agents to prevent cardiovascular diseases.

## Figures and Tables

**Table 1:** Summary of the antioxidant capacities of huskless barley *in vitro*.

Sample		ABTS		FRAP (*μ*mol FeSO_4_/g DW)	ORAC (*μ*mol TE/g DW)
		IC_50_ (g/L)	TEAC (mg/g DW)		
Black and white huskless barley (BW80)	AE	2.01 ± 0.06^b^	9.18 ± 0.09^d^	84.1 ± 1.98^e^	652.45 ± 13.03^a^
	WE	9.91 ± 0.09^h,i^	1.86 ± 0.04^k^	36.7 ± 0.70^i^	396.57 ± 7.16^e^

JiuGe (JG)	AE	2.22 ± 0.14^c^	8.32 ± 0.09^f^	131.1 ± 3.63^a^	539.96 ± 3.60^b^
	WE	9.68 ± 0.16^h^	1.91 ± 0.05^j,k^	26.5 ± 1.95^k^	237.35 ± 9.51^i^

DiQing 3 (DQ3)	AE	2.84 ± 0.07^e^	6.50 ± 0.18^h^	71.6 ± 1.35^f^	147.81 ± 8.50^l,m^
	WE	11.41 ± 0.11^k^	1.62 ± 0.08^l^	15.8 ± 0.75^m^	82.71 ± 2.50^n^

Long and black huskless barley (LB)	AE	2.10 ± 0.22^b,c^	8.79 ± 0.08^e^	51.1 ± 1.36^g^	205.54 ± 9.57^j^
	WE	11.74 ± 0.09^l^	1.57 ± 0.04^l^	41.6 ± 0.93^h^	127.78 ± 8.00^m^

YunQing 2 (YQ2)	AE	1.93 ± 0.04^b^	9.56 ± 0.05^c^	111.1 ± 4.15^b^	515.44 ± 4.01^c^
	WE	7.41 ± 0.08^f^	2.49 ± 0.04^i^	41.8 ± 1.41^h^	348.24 ± 13.56^f^

GuiBaDingGeNa (GBDGN)	AE	1.74 ± 0.14^a^	10.61 ± 0.10^a^	92.1 ± 2.52^d^	467.55 ± 21.09^d^
	WE	12.17 ± 0.03^m^	1.52 ± 0.07^l^	31.6 ± 2.10^j^	268.00 ± 5.29^h^

Short and white huskless barley (SW)	AE	1.74 ± 0.15^a^	10.61 ± 0.10^a^	100.0 ± 4.10^c^	322.41 ± 11.00^g^
	WE	10.26 ± 0.05^j^	1.80 ± 0.06^k^	34.9 ± 1.92^i,j^	71.49 ± 4.50^o^

QingHaiHuang (QHH)	AE	2.58 ± 0.07^d^	7.16 ± 0.03^g^	81.4 ± 2.62^e^	248.89 ± 11.05^h,i^
	WE	13.43 ± 0.14^n^	1.37 ± 0.05^m^	41.8 ± 1.70^h^	153.67 ± 9.58^l^

NanLongGeNa (NLGN)	AE	1.90 ± 0.03^a,b^	9.72 ± 0.075^b^	97.2 ± 3.46^c,d^	332.49 ± 4.08^f,g^
	WE	9.30 ± 0.08^g^	1.98 ± 0.055^j^	18.6 ± 1.75^l^	172.56 ± 7.17^k^

AE: alkaline extract; WE: water extract. Different letters in the same column indicate significant differences (*P* < 0.05, as indicated by Fisher's least significant difference (LSD) test).

## Abbreviations

AAPH: 2,2′-Azobis(2-methylpropionamide) dihydrochloride
ABTS: 2,2-Azino-bis(3-ethylbenzothiazoline-6-sulfonic acid) diammonium salt
ACE: Angiotensin-converting enzyme
AE: Alkaline extracted polysaccharide
Ang II: Angiotensin II
ANOVA: Analysis of variance
AOAC: Official analytical chemists
AS: Atherosclerosis
AT1: Ang II receptor 1
AX: Arabinoxylans
BW80: Black and white huskless barley 80 days
CAA: Cellular antioxidant activity
DCFH-DA: 2′,7′-Dichlorodihydrofluorescein diacetate
DMSO: Dimethylsulfoxide
DPPH: (1,1-Diphenyl-2-picrylhydrazyl radical 2,2-diphenyl-1-(2,4,6-trinitrophenyl)hydrazyl)
DQ3: DiQing 3
DW: Dried weight
ecSOD: Extracellular superoxide dismutase
ELISA: Enzyme-linked immunosorbent assay
FRAP: Ferric reducing antioxidant power
GBDGN: GuiBaDingGeNa
HUVEC: Human umbilical vein endothelial cells
IC_50_: Half maximal inhibitory concentration
JG: JiuGe
LB: Long and black huskless barley
MCP-1: Monocyte chemotactic protein 1
MTT: 3-(4,5-Dimethyl-2-thiazolyl)-2,5-diphenyl-2-H-tetrazolium bromide
NLGN: NanLongGeNa
NO: Nitric oxide
OD: Optical density
ORAC: Oxygen radical absorbance capacity
PBS: Phosphate-buffered saline
QHH: QingHaiHuang
ROS: Reactive oxygen species
SD: Standard deviation
SOD: Superoxide dismutase
SW: Short and white huskless barley
TE: Trolox equivalent
TEAC: Trolox equivalent antioxidant capacity
TNF-*α*: Tumor necrosis factor-alpha
VCAM-1: Vascular cell adhesion molecule 1
WE: Water extracted polysaccharide
YQ2: YunQing 2.

## Supplementary Materials

Supplementary Materials Figure 1: reversed-phase high performance liquid chromatographic (HPLC) profile (*λ* = 280 nm) of mixed phenolic standards. The following 9 peaks represent chemical standards: (1) gallic acid, (2) neochlorogenic acid (3-caffeoylquinic acid), (3) chlorogenic acid (5-caffeoylquinic acid), (4) cryptochlorogenic acid (4-caffeoylquinic acid), (5) rutin (quercetin-3-*O*-rutinoside), (6) isoquercetin (quercetin-3-*O*-glucoside), (7) isochologenic acid B (3,5-dicaffeoylqunic acid), (8) astragalin (kaempferol-3-O-glucoside), and (9) isochrologenic acid C (4,5-dicaffeoylqunic acid). Figure 2: reversed-phase high performance liquid chromatographic (HPLC) profiles (*λ* = 280 nm) of the phenolic compounds from different huskless barley extracts. (A) Water extract from NanLongGeNa. (B) Water extract from QingHaiHuang. (C) Alkaline extract from NanLongGeNa. (D) Alkaline extract from QingHaiHuang. The following peaks were identified: (1) gallic acid, (3) chlorogenic acid (5-caffeoylquinic acid), (4) rutin (quercetin-3-*O*-rutinoside), (5) isochologenic acid B (3,5-dicaffeoylqunic acid), (6) astragalin (kaempferol-3-O-glucoside), and (7) isochrologenic acid C (4,5-dicaffeoylqunic acid).

## References

[1] Onat D., Brillon D., Colombo P. C., Schmidt A. M. (2011). Human vascular endothelial cells: a model system for studying vascular inflammation in diabetes and atherosclerosis.

[2] Pendyala L. K., Li J., Shinke T. (2009). Endothelium-dependent vasomotor dysfunction in pig coronary arteries with paclitaxel-eluting stents is associated with inflammation and oxidative stress.

[3] Han M., Sha X., Wu Y., Fang X. (2006). Oral absorption of ginsenoside Rb_1_ using in vitro and in vivo models.

[4] Chai H., Wang Q., Huang L., Xie T., Fu Y. (2008). Ginsenoside Rb1 inhibits tumor necrosis factor-*α*-induced vascular cell adhesion molecule-1 expression in human endothelial cells.

[5] Heinecke J. W. (1998). Oxidants and antioxidants in the pathogenesis of atherosclerosis: implications for the oxidized low density lipoprotein hypothesis.

[6] Stephens N. G., Parsons A., Brown M. J. (1996). Randomised controlled trial of vitamin E in patients with coronary disease: Cambridge Heart Antioxidant Study (CHAOS).

[7] Landmesser U., Harrison D. G., Drexler H. (2006). Oxidant stress—a major cause of reduced endothelial nitric oxide availability in cardiovascular disease.

[8] Anandh Babu P., Liu D. (2008). Green tea catechins and cardiovascular health: an update.

[9] Diplock A. T., Charuleux J. L., Crozier-Willi G. (1998). Functional food science and defence against reactive oxidative species.

[10] Huang W. Y., Davidge S. T., Wu J. (2013). Bioactive natural constituents from food sources—potential use in hypertension prevention and treatment.

[11] Sawicki C., McKay D., McKeown N., Dallal G., Chen C., Blumberg J. (2016). Phytochemical pharmacokinetics and bioactivity of oat and barley flour: a randomized crossover trial.

[12] Peerajit P., Chiewchan N., Devahastin S. (2012). Effects of pretreatment methods on health-related functional properties of high dietary fibre powder from lime residues.

[13] Ma M. M., Mu T. H. (2016). Effects of extraction methods and particle size distribution on the structural, physicochemical, and functional properties of dietary fiber from deoiled cumin.

[14] Knutsen S., Holtekjolen A. (2007). Preparation and analysis of dietary fibre constituents in whole grain from hulled and hull-less barley.

[15] Arts M. J. T. J., Dallinga J. S., Voss H.-P., Haenen G. R. M. M., Bast A. (2004). A new approach to assess the total antioxidant capacity using the TEAC assay.

[16] Benzie I. F. F., Strain J. J. (1996). The ferric reducing ability of plasma (FRAP) as a measure of “antioxidant power”: the FRAP assay.

[17] Li C., Huang W.-Y., Wang X.-N., Liu W.-X. (2013). Oxygen radical absorbance capacity of different varieties of strawberry and the antioxidant stability in storage.

[18] Sharma P., Kotari S. L. (2017). Barley: impact of processing on physicochemical and thermal properties—a review.

[19] Djurle S., Andersson A. A. M., Andersson R. (2016). Milling and extrusion of six barley varieties, effects on dietary fibre and starch content and composition.

[20] Zhu Y., Li T., Fu X., Abbasi A. M., Zheng B., Liu R. H. (2015). Phenolics content, antioxidant and antiproliferative activities of dehulled highland barley (*Hordeum vulgare* L.).

[21] Zhu F., Du B., Xu B. (2015). Superfine grinding improves functional properties and antioxidant capacities of bran dietary fibre from Qingke (hull-less barley) grown in Qinghai-Tibet Plateau, China.

[22] Ueda Y., Uehara N., Sasaki H., Kobayashi K., Yamakawa T. (2013). Impacts of acute ozone stress on superoxide dismutase (SOD) expression and reactive oxygen species (ROS) formation in rice leaves.

[23] Gill S. S., Tuteja N. (2010). Reactive oxygen species and antioxidant machinery in abiotic stress tolerance in crop plants.

[24] Erdmann K., Grosser N., Schipporeit K., Schröder H. (2006). The ACE inhibitory dipeptide Met-Tyr diminishes free radical formation in human endothelial cells via induction of heme oxygenase-1 and ferritin.

[25] Sen Raychaudhuri S., Deng X. W. (2000). The role of superoxide dismutase in combating oxidative stress in higher plants.

[26] Moskovitz J., Yim M. B., Chock P. B. (2002). Free radicals and disease.

[27] Simon H.-U., Haj-Yehia A., Levi-Schaffer F. (2000). Role of reactive oxygen species (ROS) in apoptosis induction.

[28] Pelicano H., Carney D., Huang P. (2004). ROS stress in cancer cells and therapeutic implications.

[29] Koch A. E., Kunkel S. L., Harlow L. A. (1992). Enhanced production of monocyte chemoattractant protein-1 in rheumatoid arthritis.

[30] Cushing S. D., Berliner J. A., Valente A. J. (1990). Minimally modified low density lipoprotein induces monocyte chemotactic protein 1 in human endothelial cells and smooth muscle cells.

[31] Salcedo R., Ponce M. L., Young H. A. (2000). Human endothelial cells express CCR2 and respond to MCP-1: direct role of MCP-1 in angiogenesis and tumor progression.

[32] Chen X. L., Zhang Q., Zhao R., Medford R. M. (2004). Superoxide, H_2_O_2_, and iron are required for TNF-*α*-induced MCP-1 gene expression in endothelial cells: role of Rac1 and NADPH oxidase.

[33] Cybulsky M. I., Iiyama K., Li H. (2001). A major role for VCAM-1, but not ICAM-1, in early atherosclerosis.

[34] Cook-Mills J. M., Deem T. L. (2005). Active participation of endothelial cells in inflammation.

[35] Li G. H., Qu M. R., Wan J. Z., You J. M. (2007). Antihypertensive effect of rice protein hydrolysate with in vitro angiotensin I-converting enzyme inhibitory activity in spontaneously hypertensive rats.

[36] Brasier A. R., Recinos A., Eledrisi M. S. (2002). Vascular inflammation and the renin-angiotensin system.

[37] Schmidt C. G., Gonçalves L. M., Prietto L., Hackbart H. S., Furlong E. B. (2014). Antioxidant activity and enzyme inhibition of phenolic acids from fermented rice bran with fungus *Rizhopus oryzae*.

[38] Zhang H., Zhang S., Wang J., Sun B. (2017). Wheat bran feruloyl oligosaccharides protect against AAPH-induced oxidative injury via p38MAPK/PI3K-Nrf2/Keap1-MafK pathway.

