# Supplementary material for: Protective Role of Antioxidant Huskless Barley Extracts on TNF-α-Induced Endothelial Dysfunction in Human Vascular Endothelial Cells
Source: Oxid Med Cell Longev. 2018 May 10;2018:3846029. doi: 10.1155/2018/3846029 (PMC5971280; doi:10.1155/2018/3846029)
Supplement: Supplementary Materials — Figure 1: reversed-phase high performance liquid chromatographic (HPLC) profile (λ = 280 nm) of mixed phenolic standards. The following 9 peaks represent chemical standards: (1) gallic acid, (2) neochlorogenic acid (3-caffeoylquinic acid), (3) chlorogenic acid (5-caffeoylquinic acid), (4) cryptochlorogenic acid (4-caffeoylquinic acid), (5) rutin (quercetin-3-O-rutinoside), (6) isoquercetin (quercetin-3-O-glucoside), (7) isochologenic acid B (3,5-dicaffeoylqunic acid), (8) astragalin (kaempferol-3-O-glucoside), and (9) isochrologenic acid C (4,5-dicaffeoylqunic acid). Figure 2: reversed-phase high performance liquid chromatographic (HPLC) profiles (λ = 280 nm) of the phenolic compounds from different huskless barley extracts. (A) Water extract from NanLongGeNa. (B) Water extract from QingHaiHuang. (C) Alkaline extract from NanLongGeNa. (D) Alkaline extract from QingHaiHuang. The following peaks were identified: (1) gallic acid, (3) chlorogenic acid (5-caffeoylquinic acid), (4) rutin (quercetin-3-O-rutinoside), (5) isochologenic acid B (3,5-dicaffeoylqunic acid), (6) astragalin (kaempferol-3-O-glucoside), and (7) isochrologenic acid C (4,5-dicaffeoylqunic acid). [file 3846029.f1.doc]

**HPLC method**

According to *Xiao et al*. (2015), we extracted the phenolics from huskless barley extracts with methanol (80%) containing 0.1% formic acid in water bath at 50℃ for 4 h, and then centrifuged the slurry at 5000 rpm/min for 5 min at room temperature. The supernatant was collected and filtered with a syringe filter (0.22 μm PVDF membrane) for future use. We used the reversed-phase high performance liquid chromatography (HPLC) to separate the phenolic compounds of water extracts and alkaline extracts from NanLongGeNa (NLGN) and QingHaiHuang (QHH), respectively. According to the report of *Peterson et al.* (2001), phenolic solvents (5 μL) were injected onto a 250 mm×4.6 mm, 5 μm particle size, end-capped reverse-phase Zorbax SB-C18 column (Agilent Technologies), eluted over 75 min with a linear gradient of 1-40% acetonitrile with 0.1% formic acid (mobile phase A) and 99-60% water with 0.1% formic acid (mobile phase B). The DAD detection was conducted at 280 nm.


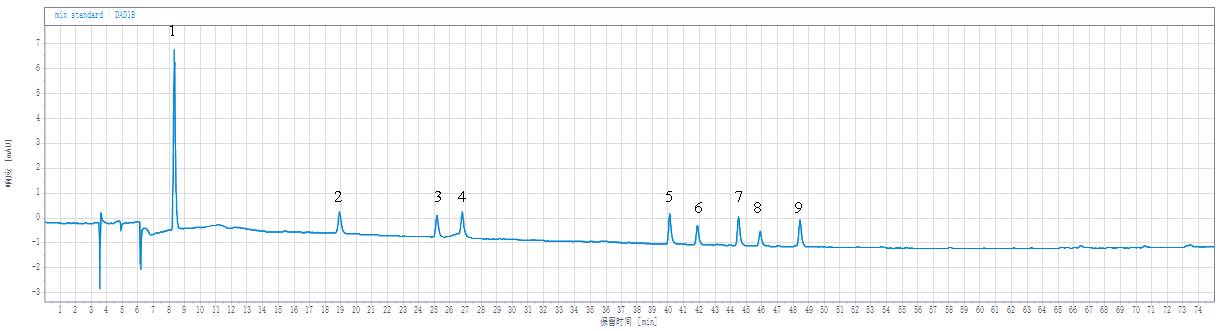
Figure 1. Reversed-phase high performance liquid chromatographic (HPLC) profile (λ = 280 nm) of mixed phenolic standards. The following 9 peaks represent chemical standards: 1. gallic acid; 2. neochlorogenic acid (3-caffeoylquinic acid); 3. chlorogenic acid (5-caffeoylquinic acid); 4. cryptochlorogenic acid (4-caffeoylquinic acid); 5. rutin (quercetin-3-*O*-rutinoside); 6. isoquercetin (quercetin-3-*O*-glucoside); 7. isochologenic acid B (3,5-dicaffeoylqunic acid); 8. astragalin (kaempferol-3-O-glucoside); 9. isochrologenic acid C (4,5-dicaffeoylqunic acid).


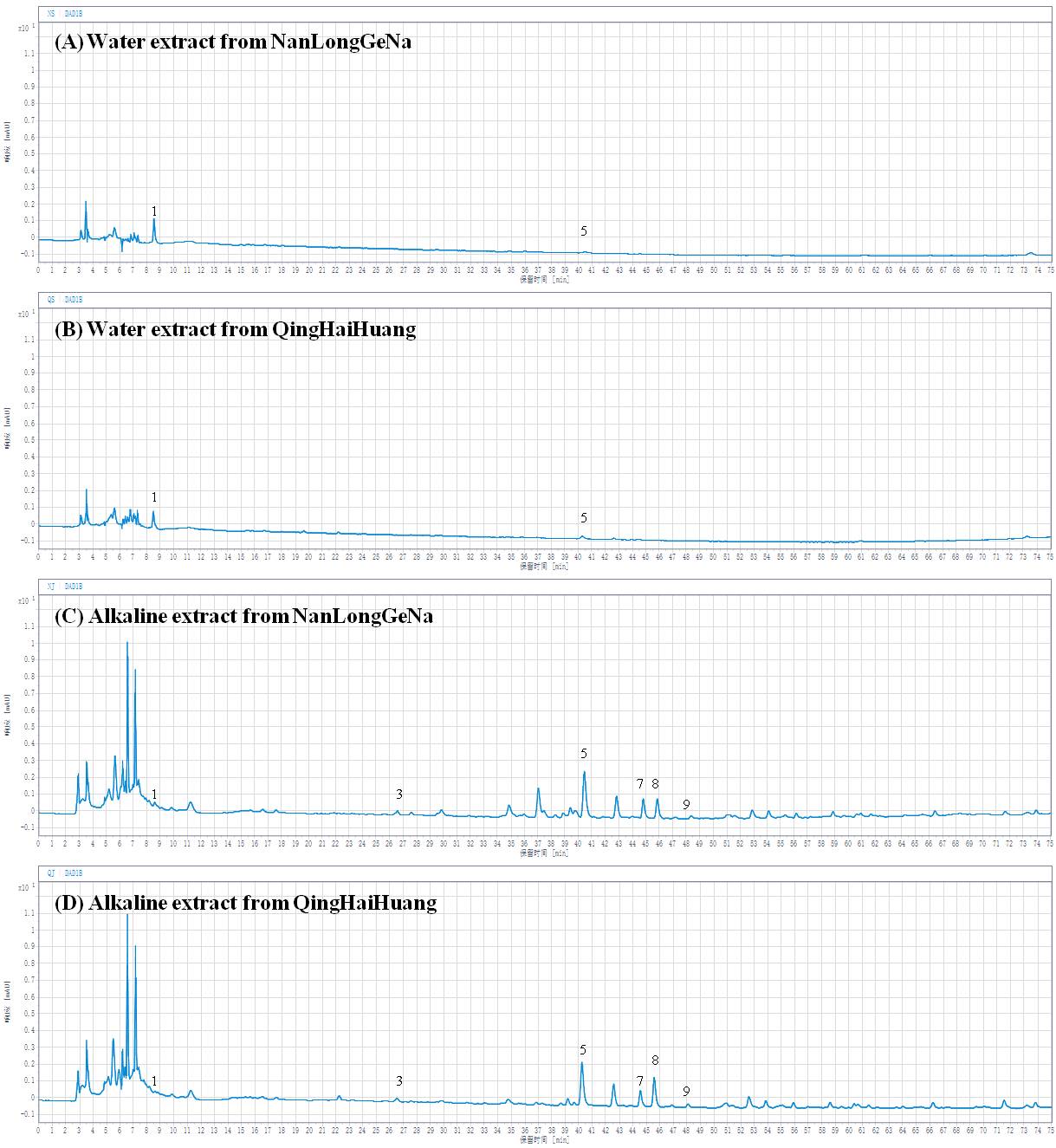


Figure 2. Reversed-phase high performance liquid chromatographic (HPLC) profiles (λ = 280 nm) of the phenolic compounds from different huskless barley extracts. (A) Water extract from NanLongGeNa; (B) Water extract from QingHaiHuang; (C) Alkaline extract from NanLongGeNa; (D) Alkaline extract from QingHaiHuang. The following peaks were identified: 1. gallic acid; 3. chlorogenic acid (5-caffeoylquinic acid); 5. rutin (quercetin-3-*O*-rutinoside); 7. isochologenic acid B (3,5-dicaffeoylqunic acid); 8. astragalin (kaempferol-3-O-glucoside); 9. isochrologenic acid C (4,5-dicaffeoylqunic acid).
